# Primary leiomyosarcoma of the small finger treated with ray amputation: A case report

**DOI:** 10.1016/j.ijscr.2023.109113

**Published:** 2023-12-07

**Authors:** Jacob Mushaben, Robin Evans

**Affiliations:** a Community Memorial Hospital, 147 Brent St., Ventura, CA 93003, United States of America; b Ventura County Medical Center, 300 Hillmont Ave, Ventura, CA 93003, United States of America

**Keywords:** Primary Leiomyosarcoma, Small finger, Ray amputation, Case report

## Abstract

**Introduction:**

Malignant sarcomas of the upper extremity are rare tumors that can have an overwhelming impact on a patient's function, quality of life, and life expectancy. Rarer still is the malignant tumor of the hand or digits, which represent an incredibly small percentage of upper extremity tumors. This paucity of cases can lead to difficult decision making and treatment options that may not always have clearly established results and outcomes.

Case.

In this case, we present a young, otherwise healthy patient that was diagnosed with a primary leiomyosarcoma of the small finger. After her diagnosis, she underwent extensive oncologic workup, and subsequently underwent successful ray amputation with an excellent outcome. She remains disease free.

**Discussion:**

Leiomyosarcoma is a malignant cancer that can be troublesome to diagnose in the extremities, as it is usually found in the smooth muscle of organs and the pelvis. Local control of the tumor is critical to successful, disease free treatment. Good functional and clinical outcomes can be attained with ray amputation, as seen in this patient.

**Conclusion:**

This case demonstrates a successful treatment approach to the patient with a primary malignant soft tissue sarcoma who was treated with a ray amputation. The clinician and surgeon must maintain a high index of suspicion of soft tissue malignancies, as a prompt diagnosis and treatment is critical to a good outcome and survival.

## Highlights

• Leiomyosarcoma of the hand and digits is a very rare malignant tumor.
• A prompt and accurate diagnosis is key to a good outcome and longer survival.
• Amputation can serve as a viable treatment option with excellent outcomes.

## 1 Introduction

Soft tissue sarcoma of the hand is a rare malignancy that can have devastating clinical and functional outcomes for patients. These tumors may represent only approximately 2 % of all soft tissue sarcomas of the upper extremities, and of this 2 % of tumors, <1 % are thought to involve the hand or fingers [1]. Often the location of the tumor or mass may hint at the possible diagnosis, and much more often than not, soft tissue masses of the hand or fingers are benign [2]. Management of soft tissue sarcomas of the hand presents a challenging treatment problem for the operating surgeon, as there is often a lack of abundant soft tissue for coverage or limb salvage available, and frequently free tissue flaps are utilized [2,3]. Attempting a limb salvage distal to the wrist could be a functional detriment to the patient due to the loss of function of the affected digit and a quadriga effect. Most commonly, local-wide excision is utilized, with amputation employed for cases that cannot be completely resected without, for recurrent disease, and when significant morbidity and compromise of hand function may result in [4,5].

Leiomyosarcoma is a malignant tumor that arises from smooth muscle cells and is most common in the smooth muscle of visceral organs and the pelvis [4]. It is exceptionally rare in the hand and fingers, with only a handful of cases reported in the literature over the past several decades [6,7]. Due to its rare occurrence, there can be a delay in diagnosis, leading to possible increases in morbidity and loss of function [7,8]. A proper diagnosis can be difficult, given its similarities to other smooth muscle soft tissue sarcomas, and prompt and proper immunohistochemical staining can be a key to diagnosis [9,10].

We present the case of a young female who underwent successful minor finger ray amputation of the right hand for a primary leiomyosarcoma of the small finger. This case will add to the very sparse literature available for the surgical treatment of leiomyosarcomas of the digits and provide a thoughtful discussion that can help guide treatment decisions in the future. This case occurred and was followed throughout at a community hospital. A consent was obtained from the patient to present this case study, which included release of photos. This report was written following the approval of the ethics committee at Community Memorial Hospital (147 N. Brent St., Ventura, CA) on July 1, 2023. This report has been reported in line with the SCARE Guidelines.

## 2 Case report

**Fig. 1:**
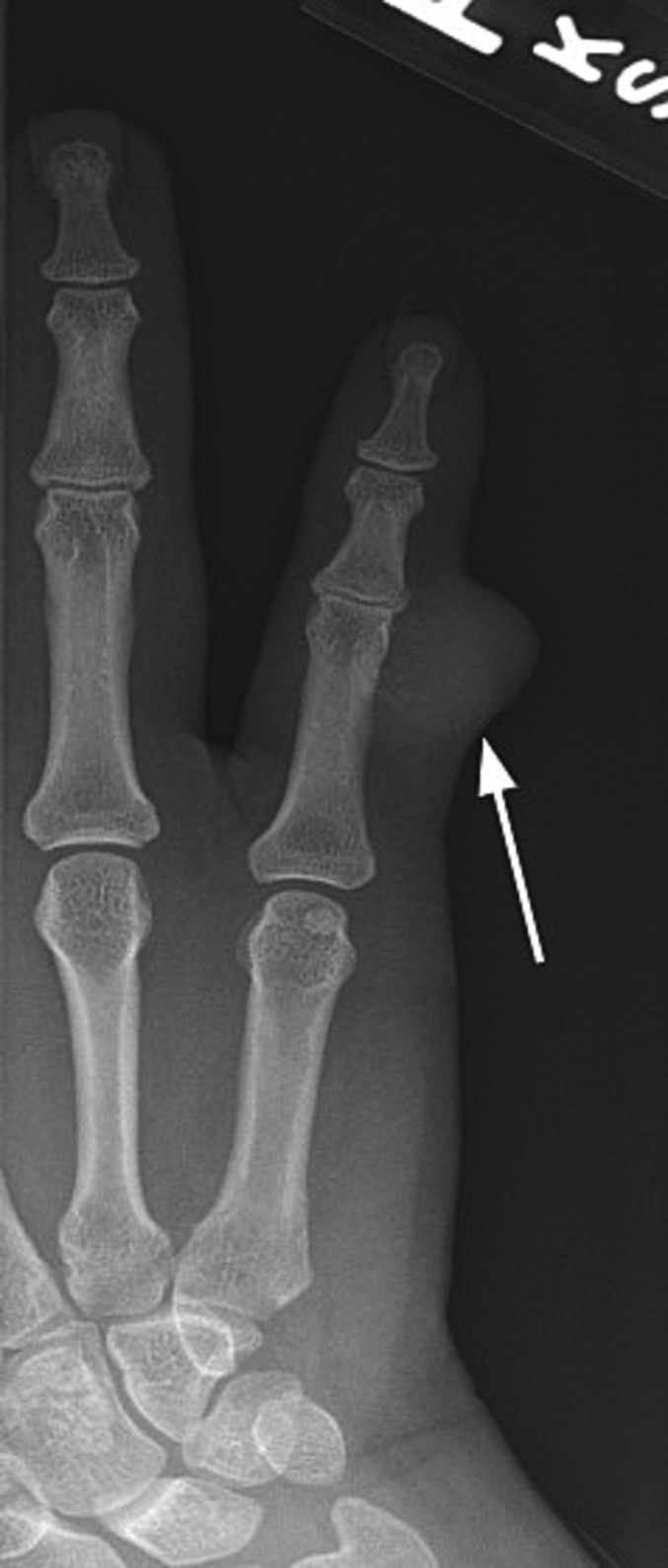
right finger x-ray showing the soft tissue mass and small calcifications present in the small finger of the patient, March 2022.

**Fig. 2:**
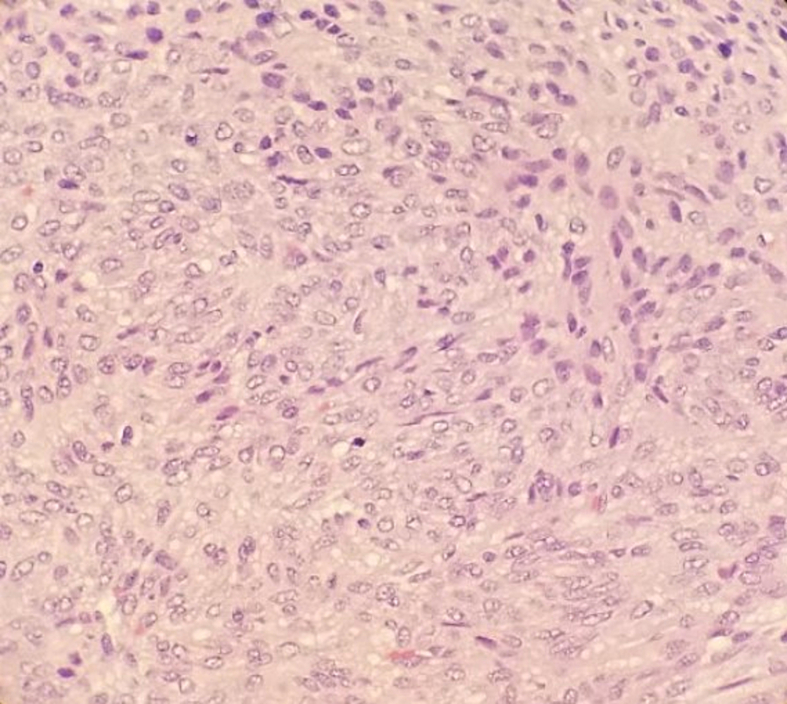
Histological specimen of the right small finger mass that was surgically excised in March 2022. Cells show classic blunt ended nuclei and many mitotic figures. Immunohistochemistry was positive for SMA, Caldesmon, and Desmin, negative for S100. This is most consistent with a soft tissue sarcoma of smooth muscle differentiation.

**Fig. 3:**
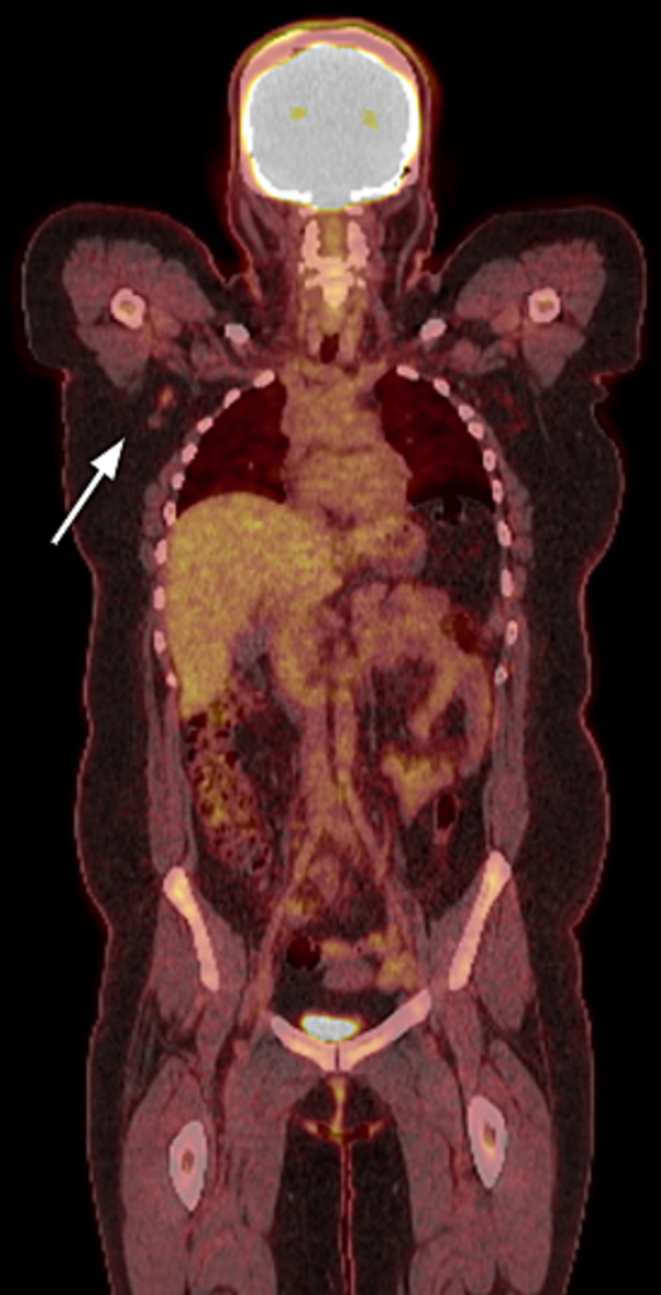
PET-CT scan of the patient in April 2022 demonstrating positive scan for a right sided axillary lymph node.

The patient is a 24-year-old right-hand dominant Hispanic female that initially presented to an urgent care office in October 2021 with a right small finger mass on the ulnar aspect of the proximal interphalangeal joint that had been present for several months prior to presentation. She had no pertinent medical or surgical history and a family history positive for renal cell carcinoma and diabetes on her maternal side of the family. She reported the mass would grow and reduce in size, especially during her pregnancy, when at times, she states it would grow to the size of an olive. Her pain worsened after pregnancy, with her newborn child grasping the finger frequently. The urgent care provider attempted aspiration of the mass, which was unsuccessful, and it was diagnosed as a likely ganglion cyst. The patient saw another local hand surgeon sometime after her urgent care visit, with apparent plans to remove the mass that was never executed. In March 2022, the patient presented to the treating hand surgeon with complaints of increasing pain and an open wound with bleeding from the original site of the mass starting one month prior. Plain radiographs (Fig. 1) demonstrated a soft tissue mass on the ulnar aspect of the small finger near the PIP joint, with some possible small punctate calcifications seen within. The patient underwent a surgical excisional biopsy of the mass on 3/15/22. The surgical pathology was reviewed by two independent pathologists, which was consistent with low-grade leiomyosarcoma of the finger. The immunohistochemical staining and histological slides are seen in Fig. 2. The patient underwent a PET/CT scan in early April 2022 (Fig. 3), demonstrating a suspicious lymph node in the right axilla. However, the treating oncologist thought it was benign and likely reactive. This is a clinical and pathologic assessment consistent with AJCC stage 1 A disease. The right small finger mass was also appreciated on the PET/CT scan. A general surgeon was also consulted for a second opinion and felt the lymph node to be benign and reactive.

**Fig. 4:**
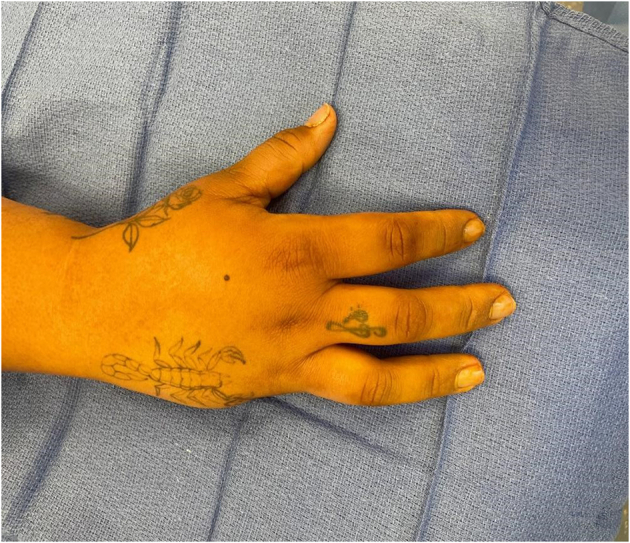
Intraoperative clinical photo of the patient, after a right small finger ray amputation.

**Figs. 5-6:**
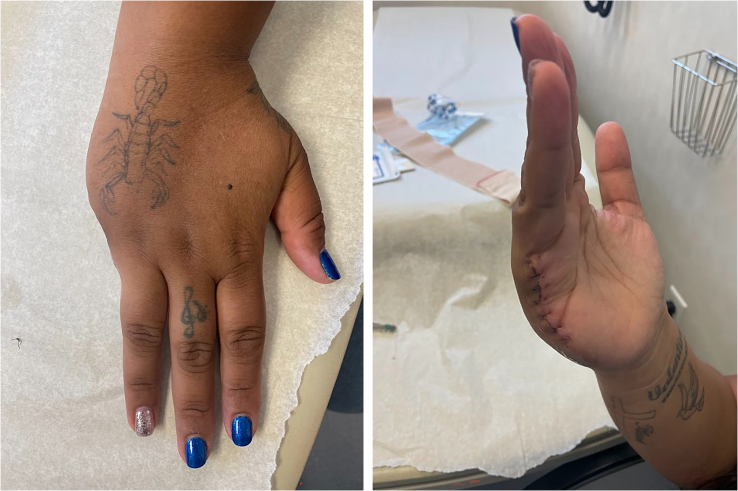
One month postoperative clinical photos of the patient, after a right small finger ray amputation.

**Figs. 7-8:**
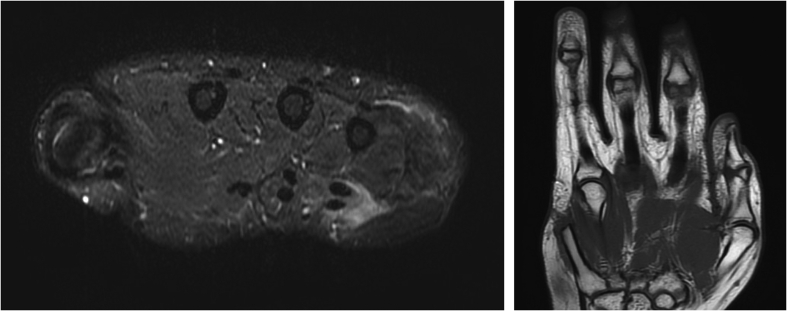
MRI of the right hand and fingers in October 2022 showing the prior small finger ray amputation, with no evidence of disease recurrence.

Given the above clinical and imaging findings, in early May 2022, the patient was discussed at the tumor board of the treating hospital, and it was agreed that she would likely get the most benefit from a ray amputation. Routine laboratory studies around this time were unremarkable for any infectious or inflammatory workup. The same day, the patient presented to the treating surgeon's office with a new complaint of another mass, which was more distal on the small finger than the original mass. The exam and radiographs seemed consistent with what was likely another focus of leiomyosarcoma. On 6/15/22, the patient underwent successful right-hand minor finger ray amputation for her primary leiomyosarcoma. The small finger ray was amputated to the level of the mid-metacarpal shaft. The small finger digital nerves also underwent targeted muscle reinnervation. The wound was closed with primary wound closure (Fig. 4). The surgical margins assessed by a pathologist were clear for any tumor, with the biopsy results again consistent with low-grade leiomyosarcoma. The patient was followed by her medical oncologist and the hand surgery team as an outpatient over the next several months. Her disease was stable, with the right axillary node showing no sign of change on repeat chest CT scans. The patient's right hand showed no evidence of recurrence or residual tumor clinically or on an MRI of the right hand obtained on 10/11/22. The patient continues progressing well postoperatively and initiated hand therapy approximately three weeks postoperatively. Most recent occupational therapy clinic notes indicated the full function of the right-hand digits 1–4, flexion 0.5 cm from the distal palmar crease, and comparable grip strength to the contralateral hand. She has no pain or other masses. Postoperatively she commented on a prominent and ropy scar, for which she also underwent aggressive therapy and massage (Figs. 5-6). Continued oncologic surveillance and repeat MRI of the right hand showed no local or systemic disease recurrence at nine months postoperatively (Figs. 7-8).

## 3 Discussion

This case demonstrates the successful treatment of a rare primary small finger malignancy in a young female with a ray amputation. This treatment represented the best surgical option for the patient, given the type of malignancy, the possibility that the tumor had spread distally already, and the best chance for local control. A proper surgical resection for local control remains the bedrock of extremity sarcoma treatment. In soft tissue sarcomas, local recurrence was initially described as high as 50 % with wide resection alone [11]. Other studies have shown that if the primary sarcoma is present in hand, patients may have more prolonged overall survival than those with soft tissue sarcomas in other locations in the extremities [12]. This may be because soft tissue sarcomas in the distal extremities undergo primary amputation faster than others that localize to the proximal extremities [13]. Additionally, leiomyosarcoma has been previously associated with lower overall survival in extremity sarcoma surgery, and the presence of residual cells in the margin correlated with worse overall survival [14]. However, different studies have shown that overall survival is not impacted by choice of primary limb salvage versus amputation, particularly in low-grade tumors [15,16]. Functionally, given the proximal and new distal masses on the digit, the patient felt better served with a primary amputation than with a limb salvage procedure. Prior studies have also demonstrated that patients can do well and have acceptable limitations and work output on the amputated hand [8,17]. It was also felt that the patient would not have a salvageable and functional digit if all necessary soft tissue and bone were removed for clear tumor margins, given the previous location of the biopsy-proven tumor and the new distal mass.

As with any case, the importance of a thorough history, physical exam, imaging, and a referral to a specialist adept at treating sarcomas of the extremities are critical to a good treatment outcome. Given the rarity of this malignancy, earlier studies have shown that there is the potential for a delay in diagnosis and worse outcomes [6]. This patient was initially misdiagnosed with a cyst and had an incorrect workup and diagnosis for several months before being referred to a specialist and hand surgeon. As has been previously discussed, the immunohistochemical analysis of the surgically excised specimen was key to an accurate diagnosis. After the initial surgical biopsy and pathologic diagnosis, the patient was presented at the multidisciplinary tumor board at the treating hospital. A recent multicenter study showed that this could lead to improved outcomes and longer relapse-free survival in these patients [18]. Routine follow up to monitor for local disease recurrence and distant metastasis is also a vital part of the care for any patient with soft tissue sarcoma. Local recurrence for extremity leiomyosarcomas can occur at a rate of up to 4 % past five years [19]. However, a recent prospective study has indicated that follow up intervals of less than six months for low-grade tumors are unlikely to yield any increased benefit, and any follow up past ten years is likely unnecessary [20]. To the date of this report, the patient has not had any local or systemic recurrence, and she will be followed closely by the treating oncologic specialist at their clinical discretion.

## 4 Conclusion

Leiomyosarcoma of the extremity is a rare malignancy, often treated with wide surgical excision, limb salvage, and, in some cases, adjuvant radiation. Very rare is the presence of this malignant tumor distal to the wrist, and even rare to present primarily in the digits. This case covers the presentation, diagnosis, clinical evaluation, and workup of a single patient diagnosed with a low-grade leiomyosarcoma of the small finger that was eventually successfully treated with primary ray amputation. It highlights the necessity of the surgeon to have a high clinical index of suspicion for any hand mass and to obtain a proper diagnosis before proceeding with treatment. It also emphasizes using primary ray amputation as a viable and successful treatment option for malignant digit tumors, with good clinical and radiographic outcomes.

## Ethical approval

Approval was granted by the sponsoring institution(s) ethics committee.

## Funding

None.

## Author contribution

Dr. Jacob Mushaben – data collection, data analysis, writing of the paper.

Dr. Robin Evans – study concept, data collection.

## Guarantor

Robin Evans.

## Research registration number

Does not apply; not a first in man study/report.

## Consent

Written and verbal consent for this report attained from the patient prior to publication.

## Declaration of competing interest

None.
